# Transparent Wood Biocomposite of Well-Dispersed Dye Content for Fluorescence and Lasing Applications

**DOI:** 10.1021/acsaom.3c00100

**Published:** 2023-05-15

**Authors:** Martin Höglund, Adil Baitenov, Lars A. Berglund, Sergei Popov

**Affiliations:** † Department of Fibre and Polymer Technology, Wallenberg Wood Science Center, KTH Royal Institute of Technology, Stockholm SE-100 44, Sweden; ‡ Department of Applied Physics, KTH Royal Institute of Technology, Stockholm 114 19, Sweden

**Keywords:** solid-state dye lasers, wood laser, random lasing, nanocomposite, delignified wood

## Abstract

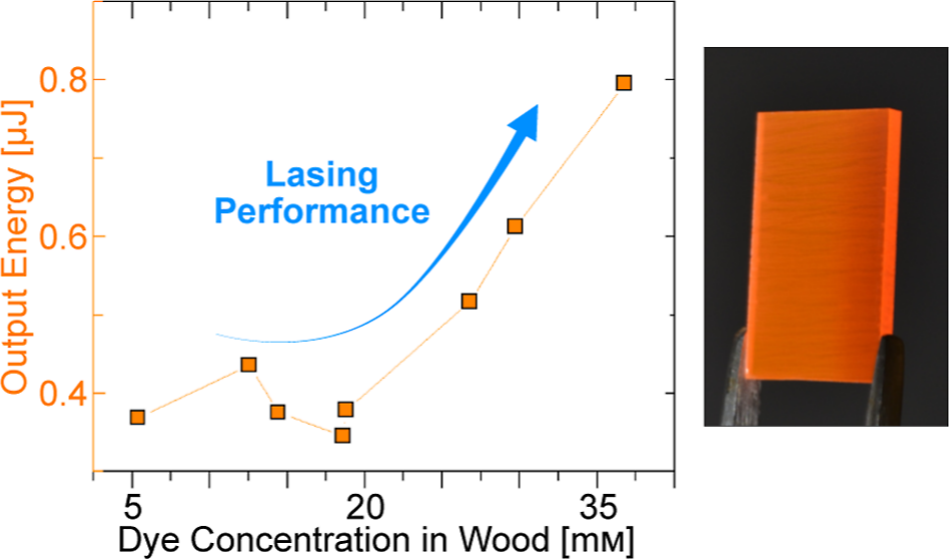

Aggregation-induced quenching often restricts emissive performance of optically active solid materials with embedded fluorescent dyes. Delignified and nanoporous wood readily adsorbs organic dyes and is investigated as a host material for rhodamine 6G (R6G). High concentration of R6G (>35 mM) is achieved in delignified wood without any ground-state dye aggregation. To evaluate emissive performance, a solid-state random dye laser is prepared using the dye-doped wood substrates. The performance in terms of lasing threshold and efficiency was improved with increased dye content due to the ability of delignified wood to disperse R6G.

## Introduction

Fluorescent materials find a broad range of applications in sensors,^1^ optoelectronics,^2^ biomedicine,^3^ and lasers.^4,5^ Aggregation-induced quenching of fluorescence, however, limits the use of highly concentrated dyes in solid materials. Dye concentrations seldom exceed 1.5 mM in polymers.^6,7^ Recently, high dye concentrations with low quenching were achieved by the use of small-molecule ionic isolation lattices^8^ or counter-ion exchange.^9^ Adsorption of fluorophores to solid substrates in guest–host systems offers an alternative approach.^10−12^ Previous research has demonstrated increased dye concentrations by adsorbing dyes onto silica,^13^ microcrystalline cellulose,^14^ and silk fibroin,^15^ although often at the expense of emissive performance.

Wood is a porous and cellular solid which has evolved so that the tree can transport and absorb liquids and nutrients from the soil,^16^ suggesting that wood may be a suitable host material for fluorescent dyes. Delignification of wood removes chromophores and makes the wood cell walls highly absorptive by increasing specific surface area. Such substrates have produced wood sponges with high oil absorption.^17,18^ Furthermore, transparent wood biocomposites (TW) suitable for optical devices can be produced from delignified wood by filling pore space with a polymer matrix.^19,20^ The hierarchical structure of wood, with many levels of order ranging from nano- to macroscale, provides intriguing optical properties in TW, high optical transmittance with high^19,21^ to low haze^22−24^ (the fraction of forward-scattered light), wave-guiding,^25,26^ anisotropic scattering,^27^ and polarization.^28^ The mechanisms of photon transport in TW have been characterized by a combined theoretical and experimental approach.^29^ The hierarchical structure promotes optical scattering, including Rayleigh scattering, by provision of many interfaces at different scales.^29^

Inclusion of additives provides TW with novel optical properties: IR-shielding from nanoparticles,^30^ structural coloring from plasmons,^31^ light filtering,^32^ and fluorescence^25^ from quantum dots. A TW-based solid-state dye laser was previously demonstrated by the incorporation of the fluorescent dye rhodamine 6G (R6G).^33^ Conventional dye lasers offer broadband tunability with narrow linewidth,^4^ biocompatibility,^5^ and the possibility to produce highly coherent,^34^ random,^35,36^ or circularly polarized lasing emission.^37^ In the case of TW, wood is both a host for the dye and a light scattering material, making it a good candidate for a random lasing medium. Sensors for explosive substances^38^ and speckle-free laser imaging^39^ have been demonstrated using random lasing. A unique aspect of the wood structure is the many tubular and parallel cells oriented along the growth direction of the tree. For wood lasing, the separate cells function as semi-ordered individual laser cavities in dye-doped TW. The semi-ordered structure results in increased spatial light coherence with such lasers.^33,40^ This is in contrast to most investigations of random lasers which have been studied in fully random media, such as in powders,^41,42^ suspensions,^43,44^ and polymers^6,35,45^ containing scattering particles or in in-plane randomized media, such as paper^46^ and silk films.^15^ The structure of highly aligned, tubular fibers in wood combined with the optical scattering in TW produces a laser with characteristics of both random and cavity lasers previously termed a quasi-random laser.^33,40^ Similar lasers, termed random lasers with non-distributed feedback, have previously been demonstrated by placing a gain medium between two rough surfaces that scatter light.^47,48^ Hybrid lasers have also been prepared using a rough surface and a mirror.^49^

In this work, we investigate delignified wood with adsorbed and highly concentrated R6G dye and we evaluate effects on the lasing performance of dye-doped TW. The present TW-based guest–host lasing material can have multi-fold increased dye concentration without the formation of aggregated clusters. As a result, the lasing performance, in terms of efficiency and lasing threshold, improves with higher dye content.

## Results and Discussion

### Material Preparation

**Figure 1:**
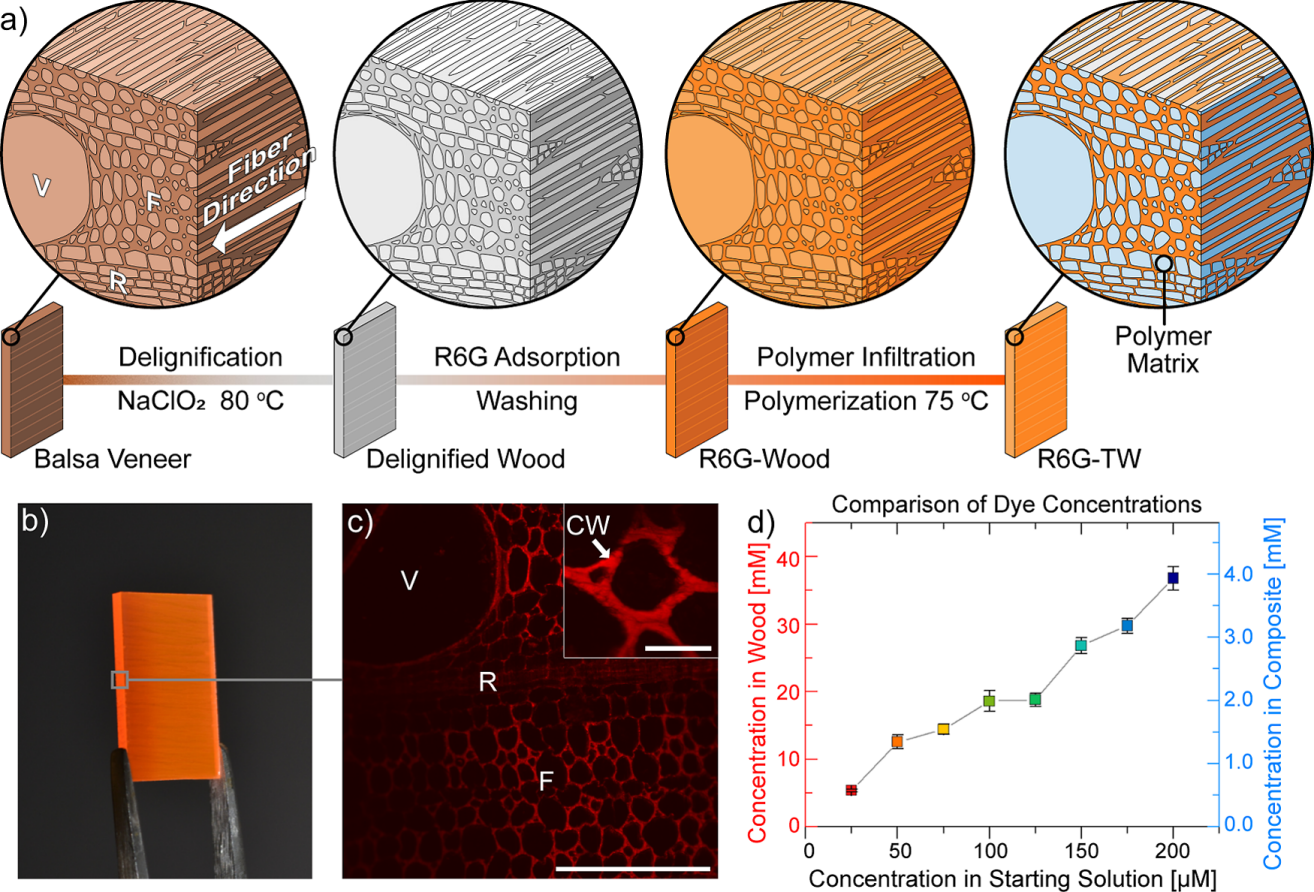
(a) Preparation of dye-doped transparent wood biocomposites, R6G-TW. Illustrations of the anisotropic wood structure. Fibers, rays, and vessels are marked as F, R, and V, respectively. In the R6G-TW structure at the far right, the polymer matrix (blue) and dye (orange) distributions are illustrated. (b) Photograph of R6G-TW of 26.8 mM dye concentration in wood. The gray square indicates the cross section of the R6G-TW structure. (c) Cross-sectional confocal fluorescence micrographs of R6G-TW of 36.8 mM dye concentration in wood. V, R, F, and CW refer to vessel, rays, fibers, and cell wall, respectively. The scale bar represents 200 μm. The inset shows a higher magnification micrograph, with a scale bar of 20 μm. (d) Comparative graph of R6G dye concentration in wood and in the composite, versus starting concentration in solution (average of three samples).

Figure 1a illustrates the preparation steps of transparent wood biocomposites doped with R6G (R6G-TW), and Figure 1b shows a photograph of a finished sample. Wood chromophores, primarily in lignin, were first removed by delignification. Delignified wood has increased porosity,^18^ specific surface area,^19,50^ and hygroscopicity^51^ compared to native wood, potentially improving its capacity for dye adsorption. R6G was adsorbed by submerging delignified wood in acetone solutions containing different amounts of the dye, with concentrations ranging from 25 to 200 μM. R6G-wood was then thoroughly washed with acetone to remove excess dye. Pre-polymerized oligomers of methyl(methacrylate) were infiltrated and then solidified by thermal polymerization. The final R6G-TW samples exhibited a strong orange hue from R6G and high transmittance of light (74% transmittance at 550 nm for reference TW, Figure S1).

The structure of balsa wood (shown in Figure 1a) mainly consists of highly aligned tubular fibers (5–35 μm width and 500–1000 μm length) providing stiffness and strength, with large open-ended vessels (150–250 μm width) for liquid transport, and small ray cells (5–35 μm width and 20–80 μm length) and similarly sized axial parenchyma cells for storage of nutrients. Vessels are oriented along the fibers, while ray cells are oriented radially in the tree stem, perpendicular to the fibers. In R6G-TW, the dye showed a strongly preferred distribution in the wood cell wall, based on confocal fluorescence micrographs (Figure 1c). The dye was distributed homogeneously inside the cell walls (1–3 μm thick) throughout the wood structure, with negligible amounts of dye in the PMMA polymer matrix (Figure S2). Although the PMMA polymer matrix has filled the void space inside the wood cells of R6G-TW, as pictured in Figure 1a, the matrix is not visible in the fluorescence micrograph (Figure 1c) since it does not contain fluorescing dye. Specimens are described by the concentration of dye in the wood component since the dye is mainly distributed there. The local concentrations of dye in the cell walls were estimated by assuming that all the dye that was removed from the starting dye solution was either adsorbed onto the wood cell walls or washed out. The amount of dye in the wood cell walls could be calculated by measuring the dye contents in the starting and washing solutions. The dye concentration in the cell walls was calculated from the adsorbed dye content and the cell wall volume, which was calculated from average cell wall densities, measured by pycnometry, of delignified balsa wood. The procedure is described in detail in the Supporting Information. Local concentrations of dye in wood were estimated to range from 5.4 to 36.8 mM (Figure 1d) between the different samples. This concentration is significantly higher than what has typically been reported for other solids containing well-dispersed R6G dye.^10−15^

Note that the dye concentrations in the cell walls do not represent the overall dye concentrations in the transparent wood composites (Figure 1d) since wood constitutes 10 vol % of the composites. Concentrations of dye in the wood structure are however referred to for analysis of optical function, since the dye is adsorbed onto the wood structure and the local concentration of the dye describes the environment of the dye.

### Dye Dispersion

**Figure 2:**
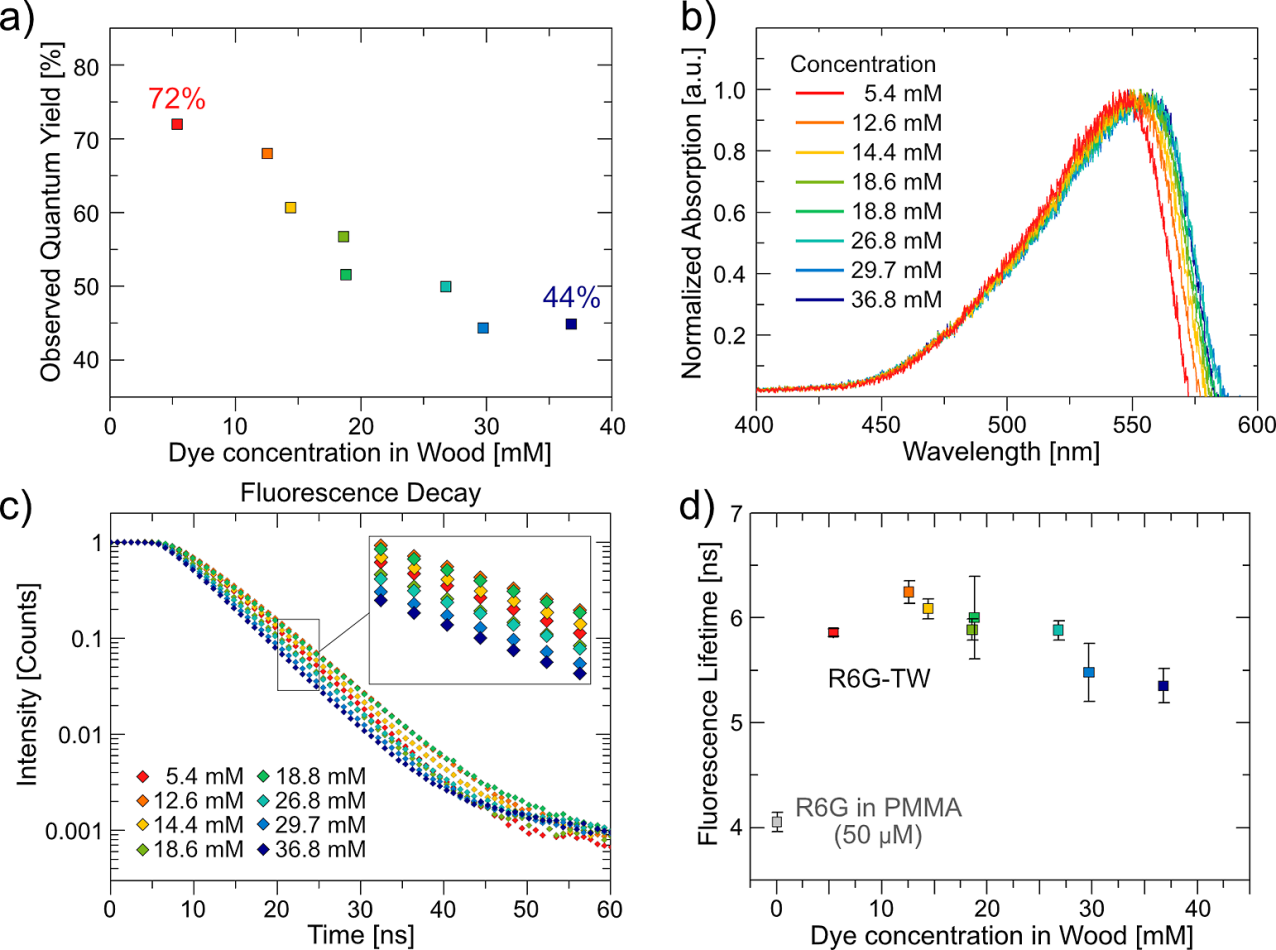
(a) Observed fluorescence QY of R6G-TW samples versus dye concentration in wood. (b) Normalized absorption spectra of R6G-TW samples. (c) Characteristic fluorescence decay curves of R6G-TW. The inset shows magnification of the marked area. (d) Fluorescence lifetimes of R6G-TW (average of six measurements).

The dispersion of the dye was investigated since high concentrations of dye can result in aggregation-induced fluorescence-quenching effects.^52^ The observed quantum yield (QY) of R6G-TW (Figure 2a), measured by diffused light illumination in an integrating sphere, shows that the QY drops from 72 to 44% with increased dye content. The loss in QY could be associated with aggregation-induced quenching, formation of statistical traps, and/or losses from inner filter effects.^52,53^ It is preferable to adjust observed QY measurements for inner filter effects to obtain the true QY of the dye in the system, but no methods are currently established for highly concentrated fluorescent materials with anisotropic scattering,^54^ such as R6G-TW. The discussion will therefore refer to observed QY instead.

Aggregation of dyes alters the electronic energy levels and quenches fluorescence by opening non-radiative relaxation pathways. The change in electronic energy levels can be distinguished by absorption spectroscopy.^55^ Absorption spectra of R6G-TW (Figure 2b) show no apparent spectral changes with increasing concentration. Specifically, no shoulder peak is present around 500–510 nm, which would be associated with dimer formation.^14,15^ It is concluded that no strong dye aggregation occurs within the investigated concentration range, meaning that delignified wood is excellent at dispersing R6G. Aromatic molecules, like R6G, tend to adsorb readily onto cellulose surfaces,^56,57^ which may explain the lack of aggregation at high concentrations of dye. There will certainly be large cellulose surfaces exposed in the nanoporous cell walls of delignified wood.

One reason for fluorescence losses without dye aggregation is statistical traps where weakly interacting monomeric dyes in close proximity generate non-radiative relaxation pathways.^52,53^ Absorption spectra are unaffected by statistical traps, but the corresponding fluorescence quenching does increase relaxation rates and decrease fluorescence lifetimes. Fluorescence decay curves are presented in Figure 2c, and the derived fluorescence lifetimes are presented in Figure 2d and Table 1. Averaged fluorescence lifetimes were estimated from the slope of the natural logarithm of exponential decay^55^
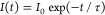
1where *I* is the measured intensity, *I*_0_ is the intensity at the time of excitation, *t* is the time in ns, and τ is the fluorescence lifetime. Averaged lifetimes are investigated due to the chemically heterogeneous structure of delignified wood (cellulose, hemicelluloses, residual lignin, etc.), which means that dye molecules will interact with a multitude of different local chemical environments. This will influence the fluorescence decay of the dye molecules and produce an ensemble of decay rates.

**Table 1:** Fluorescence Properties of Present Materials and Those Reported in the Literature: *C*_host_ (Dye Concentration in the Host Material), R6G per Host Mass (Dye Content per Weight of the Host Material), Obs. QY (Observed Quantum Yield), and τ (Fluorescence Lifetime)^b^

dye host	*C*_host_ [mM]	R6G per host mass [μmol, g^–1^]	Obs. QY [%]	τ [ns]
delignified wood (in R6G-TW)	5.4 ± 0.2	3.4 ± 0.1	72.0	5.86 ± 0.04
	12.6 ± 1.0	7.9 ± 0.7	68.0	6.25 ± 0.11
	14.4 ± 0.8	9.1 ± 0.5	60.7	6.09 ± 0.09
	18.6 ± 1.5	11.8 ± 1.0	56.7	5.89 ± 0.10
	18.8 ± 1.0	11.9 ± 0.6	51.5	6.00 ± 0.39
	26.8 ± 1.2	16.9 ± 0.7	49.9	5.88 ± 0.09
	29.7 ± 1.1	18.8 ± 0.7	44.3	5.48 ± 0.27
	36.8 ± 1.7	23.2 ± 1.1	44.9	5.35 ± 0.16
microcrystalline cellulose^14^		1.00	79^a^	3.2
		2.00	66^a^	3.0
		4.00	53^a^	2.8
silk fibroin^15^	∼16.0		10	1.2
	∼28.8		5	0.8
type II R6G-silica^13^	0.14		71	5.53
	0.5		52	5.46
	1.1		22	5.26
	1.6		21	4.17

a Fluorescence QY adjusted for inner filter effects.

b Note that “delignified wood” refers to the host (wood substrate reinforcement) combined with the polymer in the transparent wood composite R6G-TW.

The average lifetimes for R6G-TW show a peak at 6.25 ns for 12.6 mM samples and then decrease to 5.35 ns at the highest dye content. This is consistent with the formation of statistical traps when distances between dye molecules are decreasing. The dye distribution in wood is therefore non-random, as the total volume of wood would suffice to completely disperse R6G and avoid statistical traps at current concentrations.

While the formation of statistical traps lowers QY, it does not explain why the drop of QY in R6G-TW is so large. The decrease in QY is only partially explained by the formation of statistical traps. Instead, the second inner filter effect is likely to be more important, where other dye molecules reabsorb fluorescence before it leaves the material.^55^ Reabsorption leads to accumulated losses in QY since the conversion of absorbed light into fluorescence from dyes is imperfect.^52^ The high content of dye and the extended dwell time of light, due to optical scattering in R6G-TW (61% haze at 550 nm, Figure S1), make reabsorption likely. An increased amount of dye molecules increases the probability of reabsorption further since the probability of fluorescence interacting with dye molecules in their energetic ground state is increased.

Reabsorption in R6G-TW is qualitatively observed as a red shift of the absorption spectra (Figure 2b).^14,53^ The red shift takes place since emission is increasingly reabsorbed in the region overlapping the absorption band. The red shift continues with higher dye content as reabsorption increases. Reabsorption also extends the fluorescence lifetimes of the system by generating subsequent excited states. The overall lifetimes of R6G-TW are therefore higher (5.35–6.25 ns) in comparison with R6G in (non-scattering) PMMA with low concentration of dye (∼4 ns, Figure 2c). For the lowest dye contents in R6G-TW (5.4–12.6 μM), the lifetimes increased as reabsorption became more probable, but for higher contents, the lifetimes shorten instead. This is likely due to the formation of statistical traps.^53^ In summary, the dye in R6G-TW is well dispersed in the sense that no visible aggregation occurs but the QY still drops with increased dye content due to reabsorption and the formation of statistical traps.

Data for other R6G guest–host systems (Table 1) illustrate the favorable R6G dispersion in delignified wood. R6G-TW outperforms other host materials, such as silk fibroin^15^ and type II silica-R6G^13^ (R6G covalently attached to silica) at similar concentrations of R6G in terms of QY. An intriguing result is the improved QY of R6G-TW when compared with microcrystalline cellulose,^14^ since cellulose is a main component in wood. In particular since the data for microcrystalline cellulose are adjusted for inner filter effects such as reabsorption. In other words, the difference in QY relates to the environment in which individual dye molecules are located. It illustrates that the specific structure of delignified wood may be advantageous, and the high specific surface area is favorable. Cellulose microfibrils are separated in delignified wood (in the wetted state), and the interfibril distance is further increased after preparing TW.^58^ It is possible that more adsorption sites become available by the separation of cellulose microfibrils and interactions such as statistical energy trapping between dye molecules adsorbed onto fibrils are lowered.

### Lasing Performance

**Figure 3:**
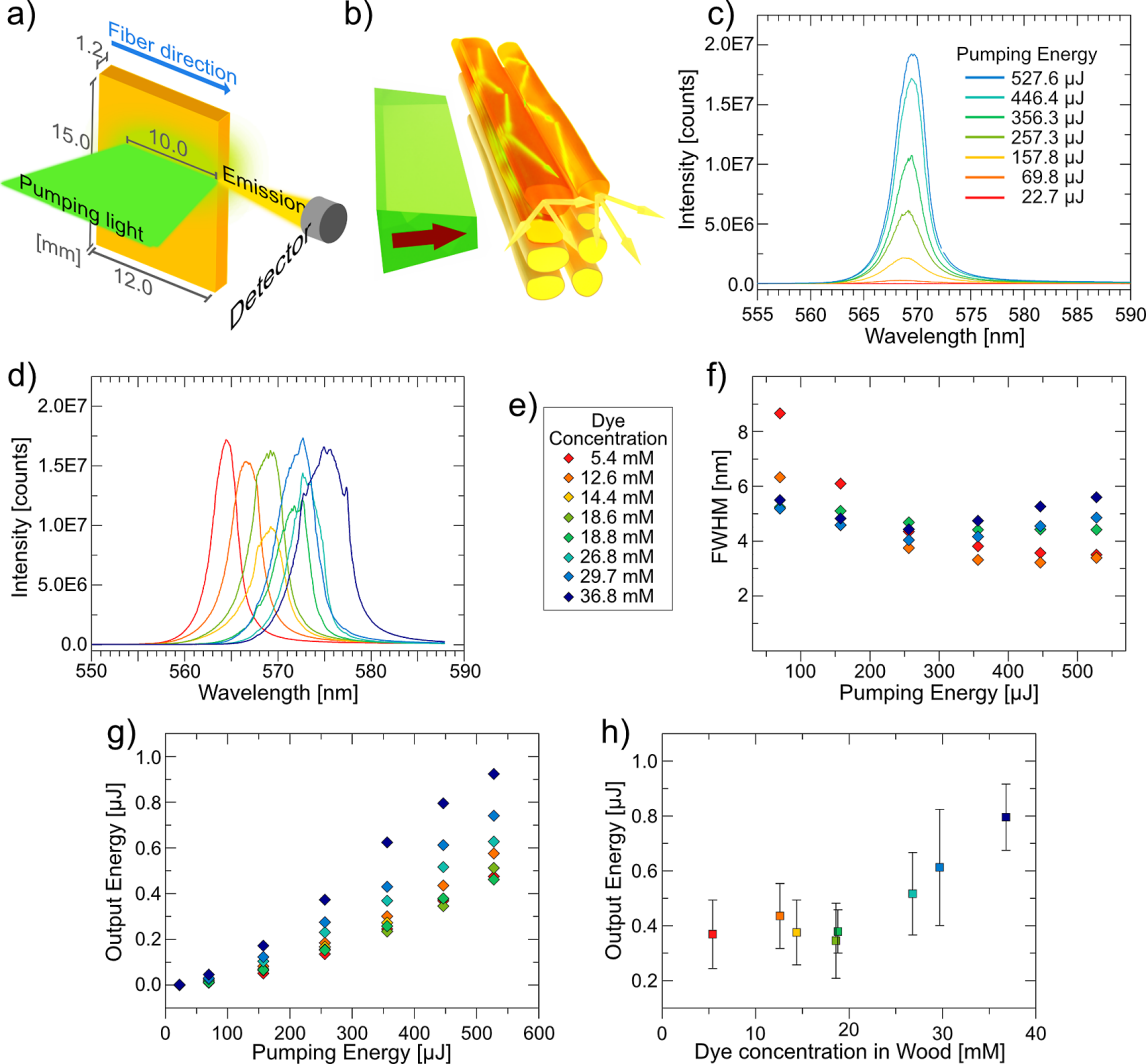
(a) Schematic layout of pumping setup for investigation of lasing properties of R6G-TW. (b) Illustration of the wave-guiding effect of wood fibers in TW. (c) Emission spectra of a 5.4 mM sample pumped with increasing pumping energies from a second harmonic generation Nd/YAG laser. (d) Characteristic lasing spectra of R6G-TW samples. (e) Color legend for graphs (d,f–h). (f) FWHM of selected R6G-TW samples as a function of pumping energy. (g) Output energy vs pumping energy of R6G-TW samples. (h) Comparison of output energy from R6G-TW samples with different dye concentrations pumped with 446 μJ (average of three samples, six positions).

To obtain lasing emission, R6G-TW samples of a rectangular shape were pumped perpendicular to the wood fiber direction with a line-shaped beam from a second harmonic generation Nd/YAG laser (532 nm). Figure 3a shows a simplified layout of the setup for high-intensity optical pumping measurements. The complete setup is provided in ref (33). Emission was collected from the sample facet transversely cut to the wood fiber direction, since the wave-guiding effect of wood fibers partially maintains light emission along the fiber direction (illustrated in Figure 3b).^25,26^ Lasing performance data are summarized in Figure 3. Key values relating material parameters and lasing performance are listed in Table 2. Note that the slope efficiency presented in Table 2 does not represent the total lasing efficiency of the materials since light is only collected from one sample facet, while the total lasing emission emanates in diverse spatial directions. This is due to R6G-TW generating random lasing radiation, rather than a conventional, spatially directed, laser beam.^59,60^ Estimation of the total lasing efficiency requires measurements from all spatial directions, which is technically very challenging.

**Table 2:** Lasing Characteristics of R6G-TW^a^

sample (dye concentration) [mM]	FWHM [nm]	centroid [nm]	threshold [μJ]	slope efficiency [%]
5.4	3.57 ± 0.54	568.6 ± 0.9	134.8	0.12
12.6	3.22 ± 0.40	572.2 ± 0.9	133.3	0.13
14.4	4.15 ± 0.71	573.6 ± 1.6	143.4	0.12
18.6	3.99 ± 0.39	573.6 ± 1.6	156.6	0.12
18.8	4.43 ± 0.39	575.8 ± 1.9	129.4	0.11
26.8	4.34 ± 0.42	577.0 ± 2.0	129.6	0.14
29.7	4.55 ± 0.53	576.5 ± 1.5	117.5	0.17
36.8	5.26 ± 0.38	577.9 ± 1.7	104.4	0.21

a TW sample designation (dye concentration), full width at half-maximum (FWHM), centroid (measure of red shift), threshold (energy) for lasing, and slope efficiency.

Under optical pumping, R6G-TW first generates enhanced fluorescence that transforms to lasing emission when the pumping energy is increased. The lasing emission combines narrow linewidth with high spectral brightness (Figure 3c). Figure 3d (with the legend in Figure 3e) shows representative emission spectra for each dye concentration. Each emission spectrum represents an ensemble of multiple modes of lasing generated by the collective effect of wood fibers acting as multiple semi-ordered individual Fabry-Perot resonators (Figure 3b).^33,40^ Since optical scattering in TW, prepared by delignified wood and PMMA, primarily occurs at interfacial gaps between the wood cell walls and the PMMA polymer matrix,^29,61^ R6G-TW can be considered a random laser with non-distributed feedback. Such lasers have previously been prepared by placing a gain medium between two rough surfaces.^47,48^ In R6G-TW, the dye-doped cell wall constitutes the gain medium placed between scattering interfacial gaps.

The emission spectra are broadened and red-shifted with increased dye concentration. The broadening is characterized by an increased full width at half-maximum (FWHM) from 3.57 to 5.26 nm (Table 2). All samples do however exhibit FWHM below 6 nm, which is distinctive for lasing from dye gain materials placed in low-quality cavities. Generally, the FWHM decreases with increased pumping energy since the emission evolves from fluorescence to lasing (Figure 3f). However, for R6G with higher dye content, FWHM broadens with higher pumping energies instead. Seemingly, more lasing modes are available in R6G-TW with higher dye content. With increased pumping power, the penetration of the pumping beam deepens and the illuminated volume grows, activating additional individual lasers and lasing modes that broaden the FWHM.

The red shift of the emission spectra can be characterized by their centroids (center of gravity of the integral of the emission spectrum). The centroids shifted from 568.6 to 577.9 nm (Table 2) with increased dye concentration. The red shift indicates increased dye–dye interactions from closer proximity to each other^52,53^ and corroborates the fluorescence lifetime data (Figure 2d and Table 1) and implies increased formation of excitation energy traps.

Lasing performance in R6G-TW is evaluated by the lasing threshold and efficiency (threshold and slope efficiency in Table 2). Both values were calculated from the output energies plotted against pumping energy (Figure 3g) after converting emission spectra to output energy. Remember that the slope efficiencies do not represent the total lasing efficiencies since the measurement setup only collects emission from one facet of the samples and not the total emission.

In general, the lasing performance of R6G-TW improves with higher dye concentration, which is apparent in Figure 3h which shows the output energy vs the dye concentration, when samples are pumped with 446 μJ. From the lowest to the highest dye concentration, the lasing threshold drops from 134.8 to 104.4 μJ and the slope efficiency improves from 0.12 to 0.21% (Table 2). In more detail, the lasing threshold first increases in the 5.4 to 18.6 mM range and then steadily drops for higher concentrations. The formation of statistical traps seemingly lowers lasing performance up to a dye concentration of 18.6 mM, but for even higher concentrations, the lasing enhancement from increased dye content appears to grow faster than the competing reduction from the formation of statistical traps. This result is rather unexpected since energy trapping is often presumed to negate improvements from increased dye content. In R6G-TW, the enhanced optical gain dominates since R6G is adsorbed onto the wood structure where it is sufficiently well dispersed to limit statistical trap formation. Delignified balsa wood can adsorb high concentrations of R6G in a well-dispersed manner.

### Why Lasing Performance Improves Despite Lowered Observed QY

**Figure 4:**
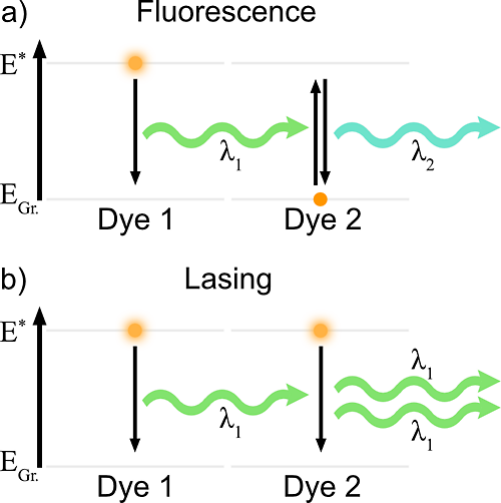
Energy diagrams showing the main excitation and radiative relaxation events for (a) fluorescence where reabsorption occurs due to dye molecules primarily populating the electronic ground state and (b) lasing where stimulated emission occurs because of population inversion (excited electronic states are primarily populated).

QY signifies the ability of a material system to convert absorbed photons into emission. Here, the lasing performance is improved with increased dye concentration, although observed QY drops, which may seem contradictory. This is related to the loss mechanism for QY data. The formation of aggregates and energy traps decreases the QY through non-radiative pathways so that emission is decreased under both fluorescence and lasing conditions. Inner filter effects, such as reabsorption and re-emission of fluorescence, also lower QY, but these effects greatly diminish during lasing due to population inversion. The mechanism is illustrated in Figure 4.

During fluorescence measurements, the majority of dye molecules populate the electronic ground state in equilibrium. When an excited dye molecule randomly relaxes and fluoresces, nearby ground state dyes are likely to absorb the fluorescence. Multiple excitation–relaxation cycles are generated with accumulative losses in emission due to imperfect conversions of absorbed energy into fluorescence by dye molecules.

To achieve lasing, high-intensity optical pumping is required to establish population inversion, a state where dye molecules primarily populate excited electronic states.^62^ Stimulated emission occurs when spontaneous emission from an excited dye molecule induces radiative relaxation in another excited dye molecule. The stimulated emission has the same wavelength and phase of optical wave as the stimulating emission. During lasing, a cascading effect of stimulated emission produces narrow linewidth emission of high intensity. The limited availability of ground-state dyes during population inversion promotes lasing properties, while reducing the extent of reabsorption.

The energetic state of the local dye population can thus explain the seeming contradiction between decreased fluorescence QY and increased lasing performance. The fluorescence QY parameter is measured under conditions where significant reabsorption can occur, whereas this mechanism is much less important during conditions of lasing. In the present R6G-TW system, with high concentrations of fluorescent dye and high extent of light scattering, reabsorption is highly probable during fluorescence QY measurements. There are methods available to adjust observed QY data for reabsorption effects, but they are not applicable for materials like R6G-TW that has anisotropic light scattering and high transmittance. Known methods require materials that are either optically thick (non-transmitting),^63^ homogeneously scattering,^64^ or show un-attenuated emission spectra (i.e., from a non-scattering sample with low dye concentration).^65^ If fluorescence QY data cannot be adjusted for reabsorption effects, such measurements becomes misleading for evaluation of potential lasing performance.

## Conclusions

Balsa wood, when delignified, is a remarkable host for R6G guest–host systems with implications for potential fluorescence and random lasing applications. The porous, nanostructured cell walls in delignified wood offer high specific surface area for dye adsorption, and their primarily cellulosic components can adsorb high local concentrations of aggregate-free R6G. In addition, the hierarchical structure of wood is helpful for dye distribution control. The fiber structure of wood functions as multiple, parallel, and tubular “optical cavities” of ≈20 μm diameter and 500–1000 μm length, which are surrounded by nanostructured cell walls, containing well-dispersed dye molecules.

Lasing transparent wood biocomposites were prepared by first adsorbing high amounts of R6G dye onto nanoporous wood substrates, followed by the incorporation of a polymer matrix. With increasing dye content, the balance between enhanced optical gain and increased non-radiative losses (from dye aggregation and energy trapping) often tilts toward losses in solid-state dye lasers, so that the optimum dye concentration becomes low. For the present R6G-TW biocomposite, however, the lasing performance steadily improved above local dye concentrations in wood of 18.6 mM. Although the formation and growth of energy trapping do occur within the investigated concentration range, the excellent dispersion of R6G keeps this limiting effect sufficiently low so that increased optical gain dominates and improves lasing performance at very high dye concentration in the wood substrate, up to at least 36.8 mM.

## Experimental Section

### Materials

Balsa wood (rotary-cut veneers, ∼209 kg, m^–3^) from Material AB (Sweden), ethanol absolute and acetone from VWR, and sodium acetate, acetic acid, sodium chlorite, R6G (laser grade 99%), methyl(methacrylate) (MMA), and azobisisobutyronitrile (AIBN) from Merck were used.

### Preparation of R6G-TW

The preparation method of transparent wood doped with R6G (R6G-TW) was adapted from refs (19) and (33).

Delignification of 20 × 10 × 1.0 mm balsa wood was performed in an acetate buffer (pH 4.6) containing 1 wt % sodium chlorite for 7 h at 80 °C. Delignified wood was subsequently washed in deionized water, and the solvent was exchanged with ethanol followed by acetone. An acetone stock solution of 200 μM R6G was used to prepare 10 mL solutions ranging from 25 to 200 μM. The stock solution was prepared by dissolving 19.4 mg of R6G in 200 mL of acetone. Each dye solution was prepared by diluting the stock solution to the desired concentrations using acetone. Single, fully acetone-wetted, delignified wood pieces were infiltrated overnight in R6G solutions. Samples were washed five times overnight in pure acetone.

Pre-polymerized PMMA was prepared by polymerizing MMA with 0.3 wt % AIBN for 30 min at 75 °C prior to sample infiltration for 4 h under vacuum. PMMA infiltrated samples were polymerized overnight at 45 °C followed by 4 h at 70 °C to ensure complete polymerization. Three samples were prepared for each concentration for statistical averaging.

### Characterization

Fluorescence micrographs were taken with confocal laser scanning microscopy performed on a Zeiss LSM 510 microscope. A 514 nm argon laser was used for excitation, and a 633 nm helium-neon laser was used for transmission imaging.

Local dye concentrations in R6G-TW wood structures were estimated from data for wood volume and the amount of adsorbed dye. The volume of wood was calculated from its mass and cell wall density of freeze-dried delignified wood. The cell wall density was measured with a Micromeritics AccuPyc 1330 pycnometer using nitrogen gas. The total adsorbed amount of dye was calculated by subtracting the amounts of dye remaining in solutions after dye adsorption and washing from the amount in the starting solution. Each solution was measured by UV/vis spectrometry performed on a Shimadzu UV-2550 spectrophotometer equipped with a 50 W halogen lamp and an R-928 photomultiplier. Scans were conducted with 0.5 nm steps and a 1 mm slit opening. The dye concentration of each solution was calculated from a calibration curve prepared with solutions of known dye concentration (Figure S3). A sample calculation for dye concentration in the wood structure can be found in the Supporting Information (Table S1).

Optical data for absorption and emission spectrometry, for observed QY, and for transmittance and haze calculations were determined using an integrating sphere with a double grating setup connected to a broadband white light lamp, a Princeton Instruments Acton SP2150, and a PIXIV100 camera. Three samples were simultaneously illuminated with diffuse light inside the integrating sphere for absorption and emission data to maximize the signal. Absorption spectra were attained by measuring samples with broadband white light. Emission spectra were measured with 500 nm illumination. Neat TW without R6G was used as the reference material. Observed QY was calculated from spectra using eq 1.
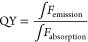
2

Transmittance and haze were measured and calculated according to ASTM D1003-03.^66^ Total and diffuse transmittance was measured by placing samples at the entrance port to the integrating sphere with either a closed or an open integrating sphere.

Fluorescence decay measurements were performed using a Zeiss LSM 510 microscope with a laser tuned to 532 nm at 20 mW power, 40 ns pulse width, and 200 ns period. The detector used was an IDQ avalanche photodiode.

The setup for high-intensity optical pumping measurements consisted of an attenuator equipped with a Nd/YAG laser (Litron Nano Series), followed by a beam expander and beam cutter with a plano convex cylindrical lens in order to create a pumping line of 10 mm width and 1 mm height on the TW sample. The signal from the sample was gathered by lenses, and spectra were measured with an Andor SR-5000-B1-R spectrometer and an Andor Zyla-4.2P-USB3-S CMOS camera with 500 ms exposure. An illustration of the setup is available in ref (33). The TW sample was pumped with 532 nm wavelength pulses with a repetition rate of 1 Hz and a pulse energy ranging from 23 to 528 μJ. Pulse energies were measured using a Thorlabs ES120C energy meter and neutral density filters. A conversion constant for converting measured counts to joules was established from the pulse energies. Integrated emission spectra were converted to emission energies using the conversion constant. It is important to take into account that the total emission energy from R6G-TW was not measured as only one of the sample facets was measured.

## Supporting Information Available

Transmittance and haze data, fluorescence micrograph with fluorescence signal intensity overlaid, and example of calculating the concentration of dye in wood structures of dye-doped transparent wood (PDF)
