# Supplementary material for: Transparent Wood Biocomposite of Well-Dispersed Dye Content for Fluorescence and Lasing Applications
Source: ACS Appl Opt Mater. 2023 May 15;1(5):1043–51. doi: 10.1021/acsaom.3c00100 (PMC10226163; doi:10.1021/acsaom.3c00100)
Supplement: Supplementary file 1 — ot3c00100_si_001.pdf [file ot3c00100_si_001.pdf]

SUPPORTING INFORMATION

Transparent wood biocomposite of well-dispersed dye content for fluorescent and lasing applications

Martin Höglund,[a] Adil Baitenov,[b] Lars A. Berglund,[a] Sergei Popov*[n]*

[a] Department of Fibre and Polymer Technology, Wallenberg Wood Science Center, KTH Royal Institute of Technology, SE-100 44 Stockholm, Sweden. E-mail: blund@kth.se

[b] Department of Applied Physics, KTH Royal Institute of Technology, 114 19 Stockholm, Sweden. E-mail: sergeip@kth.se

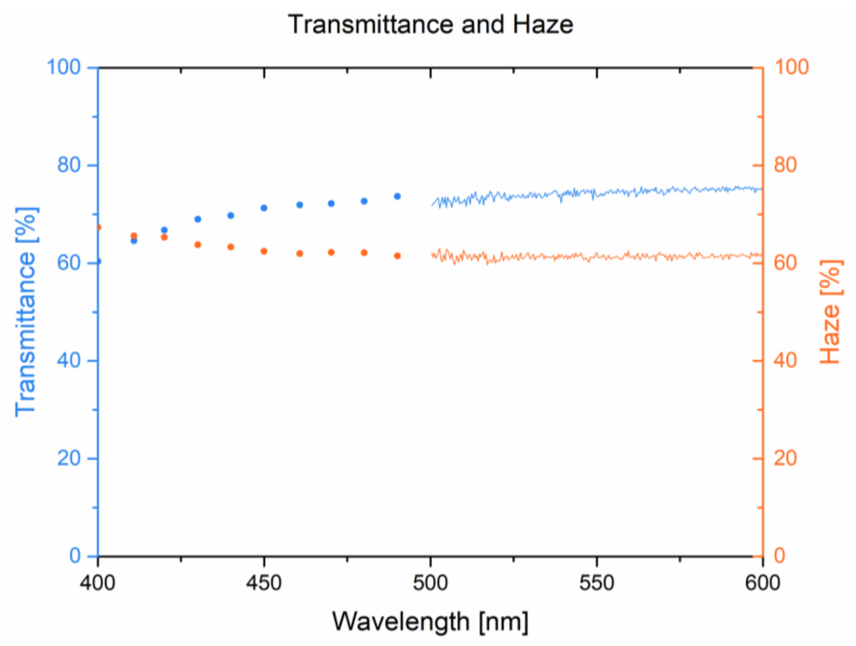

Figure S1. Transmittance (blue) and haze (orange) of transparent wood. The 500-600 nm range was measured with broadband white light. The 400-500 nm range was measured in 10 nm steps with grating selected narrow linewidth light.

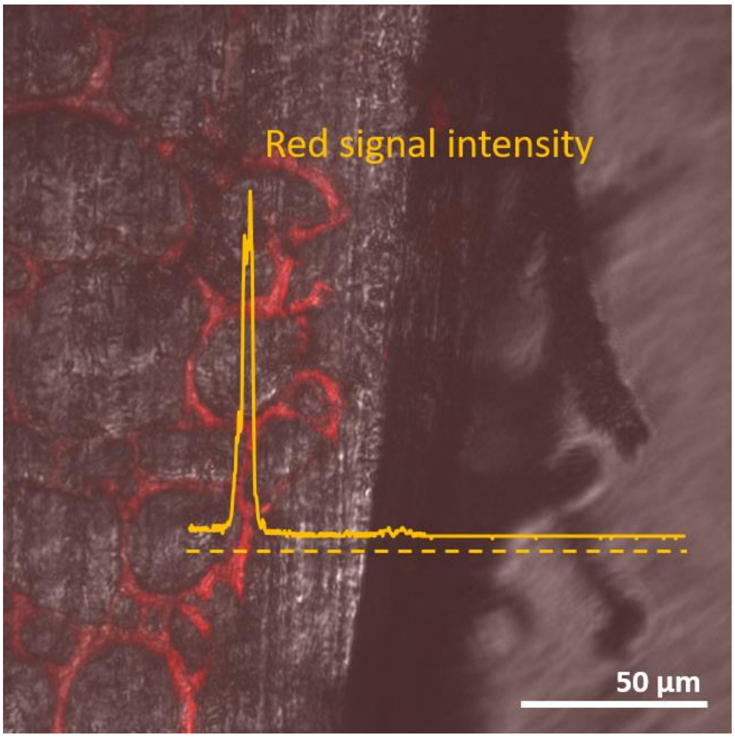

Figure S2. Composite image of CLSM fluorescence and transmission images of a 36.8 mM sample. The red signal intensity across the edge of the sample and a cell wall is overlaid. The signal show negligible amounts of dye in the polymer matrix, as it is too weak to separate from cell wall fluorescence propagating in the polymer.

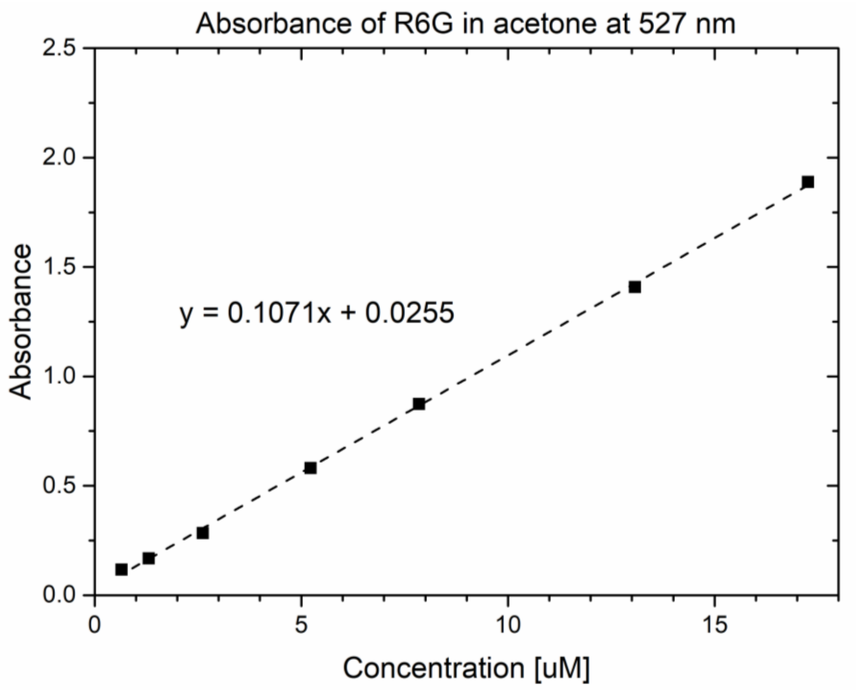

Figure S3. Calibration curve for Rhodamine 6G measured with UV/VIS spectrometry. Linear dependence of Rhodamine 6G main peak (527 nm) absorbance up to 18 μM concentration in acetone. Overlaid is the fitting (dashed line) and fitted function.

Calculation example for dye concentration in the wood structure.

The peak height of the main absorption peak (527 nm) of Rhodamine 6G (R6G) in acetone solutions (< 18 μM) was used to calculate the R6G concentration from a calibration curve (Fig. S1):

$$C_{sol.} = \frac{A_{527} - 0.0255}{0.1071} \quad \text{(S1)}$$

Where $C_{sol.}$ is the concentration of the R6G solution in μM and A_{527} is the absorbance at 527 nm. The amount of R6G in the solution can then be calculated with equation S2:

$$n_{sol.} = C_{sol.} * V_{sol.} \quad \text{(S2)}$$

Where $n_{sol.}$ is the amount of R6G in mol. The concentration inside the wood structure can then be calculated after measuring the staring solution, the starting solution after infiltration and each washing solution using equation S3:

$$C_{host} = \frac{(n_{sol.start} - (n_{sol.infltr} + n_{wash1} + \cdots + n_{wash5})) * \rho_{cellwall}}{m_{wood}} \quad \text{(S3)}$$

Where C_{host} is the concentration of R6G in the wood substrate, m_{wood} is the oven-dried weight of the substrate and $\rho_{cellwall}$ is the cell wall density of the substrate measured with pycnometry.

Table S1. Values for calculating dye concentration in wood for samples infiltrated with a 25 μM solution.

Solution	A_{527}	$C_{sol.}$ [μM]	$C_{sol.start}$ [μM]	V [ml]	$\rho_{cellwall}$ [g, cm^{-3}]	m_{wood} [mg]	C_{host} [mM]	$C_{host.avg}$ [mM]
25uM-1-w5	0.062	0.34		20				
25uM-1-w4	0.070	0.42		20				
25uM-1-w3	0.103	0.72		20				
25uM-1-w2	0.086	0.56		20				
25uM-1-w1	0.147	1.13		20				
25uM-1-sol	0.439	3.86	24.725	10	1.58	40.6	5.65	5.37 ± 0.20
25uM-2-w5	0.052	0.25		20				
25uM-2-w4	0.069	0.41		20				
25uM-2-w3	0.105	0.74		20				
25uM-2-w2	0.106	0.75		20				
25uM-2-w1	0.167	1.32		20				
25uM-2-sol	0.495	4.38	24.725	10			5.22	
25uM-3-w5	0.053	0.26		20				
25uM-3-w4	0.067	0.39		20				
25uM-3-w3	0.100	0.70		20				
25uM-3-w2	0.098	0.68		20				
25uM-3-w1	0.188	1.52		20				
25uM-3-sol	0.478	4.23	24.725	10			5.23	
